# Generation of a bank of clinical-grade, HLA-homozygous iPSC lines with high coverage of the Spanish population

**DOI:** 10.1186/s13287-023-03576-1

**Published:** 2023-12-13

**Authors:** B. Kuebler, B. Alvarez-Palomo, B. Aran, J. Castaño, L. Rodriguez, A. Raya, S. Querol Giner, A. Veiga

**Affiliations:** 1 Pluripotent Stem Cell Group, Regenerative Medicine Program, Institut d’Investigació Biomédica de Bellvitge (IDIBELL), Hospital Duran I Reynals, Gran Via de L’Hospitalet, 199-203, L’Hospitalet de Llobregat, 08908 Barcelona, Spain; 2 Program for Translation of Regenerative Medicine in Catalonia (P-[CMRC]), Hospital Duran I Reynals, Gran Via de L’Hospitalet, 199-203, L’Hospitalet de Llobregat, 08908 Barcelona, Spain; 3 Advanced and Cell Therapy Service, Banc de Sang I Teixits, Edifici Dr. Frederic Duran I Jordà, Passeig de Taulat, 106-116, 08005 Barcelona, Spain; 4 Advanced Therapy Platform, Hospital Sant Joan de Déu de Barcelona, Pg. de Sant Joan de Déu, 2, Espluges de Llobregat, 08950 Barcelona, Spain; 5 Stem Cell Potency Group, Regenerative Medicine Program, Institut d´Investigació Biomédica de Bellvitge (IDIBELL), Hospital Duran I Reynals, Gran Via de L’Hospitalet, 199-203, L’Hospitalet de Llobregat, 08908 Barcelona, Spain; 6 Centre for Networked Biomedical Research On Bioengineering, Biomaterials and Nanomedicine (CIBER-BBN), Madrid, Spain; 7 Institució Catalana de Recerca I Estudis Avançats (ICREA), Barcelona, Spain; 8 Transfusional Medicine Group, Vall d’Hebron Research Institute, Autonomous University of Barcelona (UAB), Barcelona, Spain

**Keywords:** Induced pluripotent stem cells, Haplobank, Regenerative medicine, HLA matching, GMP manufacturing

## Abstract

**Background:**

Induced pluripotent stem cell (iPSC)-derived cell therapies are an interesting new area in the field of regenerative medicine. One of the approaches to decrease the costs of iPSC-derived therapies is the use of allogenic homozygous human leukocyte antigen (HLA)-matched donors to generate iPSC lines and to build a clinical-grade iPSC bank covering a high percentage of the Spanish population.

**Methods:**

The Spanish Stem Cell Transplantation Registry was screened for cord blood units (CBUs) homozygous for the most common HLA-A, HLA-B and HLA-DRB1 haplotypes. Seven donors were selected with haplotypes covering 21.37% of the haplotypes of the Spanish population. CD34-positive hematopoietic progenitors were isolated from the mononuclear cell fraction of frozen cord blood units from each donor by density gradient centrifugation and further by immune magnetic labeling and separation using purification columns. Purified CD34 + cells were reprogrammed to iPSCs by transduction with the CTS CytoTune-iPS 2.1 Sendai Reprogramming Kit.

**Results:**

The iPSCs generated from the 7 donors were expanded, characterized, banked and registered. Master cell banks (MCBs) and working cell banks (WCBs) from the iPSCs of each donor were produced under GMP conditions in qualified clean rooms.

**Conclusions:**

Here, we present the first clinical-grade, iPSC haplobank in Spain made from CD34 + cells from seven cord blood units homozygous for the most common HLA-A, HLA-B and HLA-DRB1 haplotypes within the Spanish population. We describe their generation by transduction with Sendai viral vectors and their GMP-compliant expansion and banking. These haplolines will constitute starting materials for advanced therapy medicinal product development (ATMP).

**Supplementary Information:**

The online version contains supplementary material available at 10.1186/s13287-023-03576-1.

## Background

There is worldwide a critical need for cells and tissues for transplantation in patients with organ failure and an increasing impact of degenerative age-related human diseases for which there are very limited or no treatments available [1]. Cell therapy can constitute a future alternative to organ transplantation and for the treatment of degenerative diseases (such as macular degeneration, Parkinson's disease, heart failure, type I diabetes, or spinal cord injury, to name a few) [2] as well as a source of immune cells for cancer immunotherapy [3]. The generation of human-induced pluripotent stem cells (hiPSCs) from somatic cells offers a unique opportunity to obtain a virtually unlimited supply of a broad spectrum of specialized cells [4, 5]. hiPSC derivatives have great potential for cell replacement therapy even though the clinical relevance of such treatments is still to be realized in the form of licensed cell-based medicines [6].

The use of patient cells for the generation of hiPSCs and subsequent differentiation to the desired cell type for treatment ensures immunological compatibility and minimizes the risk of rejection. However, the time and cost necessary for the production of customized hiPSC lines and their derivatives that would be suitable for use in humans is prohibitively high.

An alternative to the use of patient-specific hiPSCs would be a hiPSC collection from allogeneic healthy donors that could be expanded and differentiated to treat different patients. To reduce the risk of immune rejection, this allogeneic hiPSC collection should comprise lines with sufficiently diverse and compatible homozygous HLA haplotypes to ensure maximum possible population coverage. hiPSC technology facilitates the prospective selection of interesting donors based on their specific HLA haplotypes [7]. Manufacturing scalable unique and standardized final cell products from homozygous haplo-selected hiPSCs suitable for various types of diseases and multiple clinical indications should reduce the cost of the final products and patient immune suppression. Cell derivatives from HLA-matched hiPSC banks will allow delivery of off-the-shelf cell therapy products that are easily accessible for critical acute or subacute diseases and for new emergent diseases such as the current pandemic SARS-2-induced inflammatory disorders.

One feasible possibility is to prospectively search for potential donors in registries/banks of bone marrow (BM) and cord blood (CB), since these donations are already typed for elements of the HLA system. Several reasons make CB cells the cell type of choice to generate homozygous HLA haplotype hiPSC collections for clinical translation: (i) there is no risk for either the mother or the new-born at collection; (ii) cord blood units, preserved in cord blood banks, are already HLA typed, which facilitates donor screening; (iii) cells in the cord blood are less likely to have accumulated genetic or epigenetic risks compared to adult and differentiated cells; and (iv) hiPSC generation methodology with CB samples is well established [8, 9]. The use of CB-hiPSCs as an alternative to the use of patient-specific hiPSCs would minimize the time and cost necessary for the production of customized hiPSCs and their derivatives. Moreover, although CB samples are designated for clinical application for hematological pathologies, many CB banks keep surplus samples sufficient to generate hiPSC lines, and CB samples with an insufficient number of hematological progenitors not suitable for transplantation might also be used.

Cell-based products for use in human therapy need to be established in GMP conditions in facilities with a relevant product manufacturing license under strict quality assurance. These products must also be generated with all ethical and legal requirements. iPSC lines are critical intermediate products for a number of emerging cell therapies [10]. The creation of hPSC banks of sufficient quality to be considered clinical grade is needed to envisage effective and safe cell therapy with such cell lines [11–13].

The generation of iPSCs acceptable for clinical use includes specificities for donor selection, original cell sourcing, reprogramming methodology, culture and expansion, testing and banking. Although some hiPSC lines have been generated in fully GMP-compliant manufacturing processes [14–16], some authors have adapted hiPSC lines established in standard conditions to expansion and banking in GMP conditions [17]. In fact, most ongoing current clinical trials are using hiPSCs generated in no-GMP conditions and then qualified for GMP by conversion in GMP conditions and additional testing [18].

Beyond full traceability of the manufacturing process, the generation of clinically compliant iPSCs requires the use of xeno-free and clinical-grade reagents as well as integration-free methods of reprogramming.

Here, we present the first clinical-grade, iPSC haplobank in Spain, generated by reprogramming CD34 + cells by Sendai viral transduction from seven cord blood units provided by the clinical inventory of the Spanish Bone Marrow registry, homozygous for the most common HLA haplotypes in Spain. The haplolines were expanded and banked with GMP-compliant methods to be used as clinical-grade intermediates for advanced therapy medicinal product development.

## Materials and methods

iPSCs were generated, expanded and frozen using xeno-free and GMP-certified or clinically approved reagents.

### Donor selection

CB units were transferred from the Spanish Bone Marrow Donor Registry (REDMO) after a reconsenting procedure using project-specific informed consent from the original donor. The ethics committee approved this procedure as part of the research project IPS-PANIA (ID ethics committee: PR(AG)428/2018). CB donors homozygous for the most frequent HLA-A, HLA-B and HLA-DRB1 haplotypes were chosen following an immunogenetic study to obtain the widest coverage of the Spanish population [19]. Homozygous CB samples had been identified in Spanish CB banks, and the selected CB units were quality verified; all participating donors signed a specific informed consent form for the use of the donated CB to generate hiPSC haplolines [20].

### Calculation of match coverage

To estimate the Spanish population HLA-matching coverage of the 7 hiPSC haplolines, we calculated the number of individuals in the population cohort with zero mismatches in HLA-A, HLA-B and HLA-DRB1 loci when compared with the haplotypes in the homozygous haplolines. The population cohort was composed of the combined data of the adult bone marrow donors and cord blood donors in the REDMO. The calculation was performed at a four-digit resolution on 56.798 individuals. The haplotype match benefit (coverage) in the whole sample was estimated with an in-house, iterative algorithm in Microsoft Access. Briefly, the first haplotype was checked against the whole cohort, and all matched individuals were extracted from the dataset. In subsequent iterations, the coverage of the following haplotype was recomputed in the remaining data. The matching was based on the concordance of the A, B and DRB1 alleles of the selected homozygous haplotypes with at least one of the two alleles of these loci in each individual. To calculate the coverage allowing for mismatch in one or two loci (beneficial match), the same strategy was applied, but one or two mismatches in any HLA loci were tolerated. In all cases, the cumulative percentage of coverage was calculated by dividing the number of matched individuals in each iteration by the total sample size, multiplied by 100.

### Isolation of cord blood progenitor cells

For CD34 + cell isolation, briefly, the cryopreserved CB units were thawed and osmotically equilibrated, and the mononuclear fraction was isolated using Ficoll in a Sepax 2 (Cytiva, Marlborough, USA) cell processor, all in a closed system. The CD34 + population was isolated with clinical-grade anti-CD34 magnetic beads (CD34 MicroBead Kit, Miltenyi Art. No. 200-070-100) and purification columns (MACS® ART MS Columns, Miltenyi, Art. No. 200-070-500), as described elsewhere [20].

### Expansion of CD34 + cells

After the extraction of CD34 + cells from cord blood units, cells were expanded in StemPro 34 Serum Free Medium (Thermo Fisher, Art. r9011) supplemented with 50 ng/ml stem cell factor (SCF, Peprotech, Art. No. GMP300-07), 50 ng/ml Fms-related tyrosine kinase 3 ligand (FLT3L, Peprotech, Art. No. GMP300-19), 10 ng/ml thrombopoietin (TPO, Peprotech, Art. No. GMP300-18) and 10 ng/ml interleukin-6 (IL-6, Peprotech, Art. No. AF-200-06) (SP34 SFM + cytokines) for 4 days at 37 $^\circ{\rm C}$ and 5% CO_2_. The medium was changed every second day.

### Generation of hiPSC lines by infection of CD34 + cells with a CTS Cytotune-iPS 2.1 reprogramming kit

After expansion, CD34 + cells were transduced with the CTS CytoTune-iPS 2.1 Sendai Reprogramming Kit (Thermo Fisher, Art. No. A34546) following the manufacturer's instructions.

Briefly, CD34 + cells were counted, and 1 × 10^4^ cells were transduced in SP34 SF medium supplemented with cytokines and 4 μg/ml polybrene at 37 $^\circ{\rm C}$ in a humidified atmosphere of 5% CO_2_ overnight. Residual CD34 + cells were cryopreserved in CTS Synth-a-Freeze Medium supplemented with CTS RevitaCell Supplement (all Thermo Fisher).

The next day, Sendai viruses were removed by centrifugation, and infected CD34 + cells were seeded on Biolaminin 521 Clinical Therapy Grade (CTG) (Biolamina, Art. No. CT521)-coated dishes in SP34 SFM supplemented with cytokines. After 3 days, the medium with floating cells was changed to SP34 SFM without cytokines by spinning. From day 6 after infection, the medium was changed to Essential 8 Flex Medium (Thermo Fisher, Art. No. A2858501), and the wells were observed every day under a stereomicroscope for the emergence of clusters of attached cells indicative of reprogrammed cells.

Approximately 16 to 18 days after transduction, single colonies were manually picked and seeded in Essential 8 Flex medium including RevitaCell supplement (CTS RevitaCell Supplement, Thermo Fisher, Art. No. A4238401) on CTG laminin-coated dishes.

After rising and picking single colonies, residual Sendai virus-infected cells were frozen in CTS Synth-a-Freeze Medium (Thermo Fisher, Art. No. A1371301) containing CTS RevitaCell Supplement (Thermo Fisher, Art. No. A4238401), and cell pellets were prepared. RNA was extracted and used as a positive control in the PCR performed to prove the absence of Sendai virus in the generated iPSC clones.

### Culture and cryopreservation of generated hiPSCs

Passaging of hiPSC clones was performed with CTS DPBS, w/o calcium, w/o magnesium (Thermo Fisher, Art. No. 1285601) and CTS Versene Solution (Thermo Fisher, Art. No. A4239101). Cells were diluted with Essential E8 Flex Medium and seeded on CTG LN-521-coated plates.

For cryopreservation, hiPSCs were centrifuged at 1000 RPM for 5 min, resuspended in 0.5 ml CTS PSC Cryomedium (Thermo Fisher, Art. No. A4238801) containing CTS RevitaCell Supplement, frozen at − 80 $^\circ{\rm C}$ in a freezing container and later transferred to a liquid nitrogen storage vessel.

### Clone selection

To select a clone for full characterization and banking, the absence of the Sendai virus genome and transgenes and, second, a normal karyotype had to be confirmed.

To verify the absence of Sendai virus in the generated hiPSCs, PCR was performed at approximately passage 7 or 8, including a culture period of 15 days at a temperature of 39 $^\circ{\rm C}$. Reprogramming of cells with Sendai virus under feeder-free conditions results in the generation of a high number of clones that are not free of virus (B. Kuebler, personal communication). Sendai virus vectors contain a temperature sensitivity mutation that causes the vectors to be removed from the generated cells when they are incubated at 39 $^\circ{\rm C}$ for 15 days.

After collecting cell pellets, total RNA was extracted following the TRIzol-based procedure and treated with DNase. As a positive control, Sendai-infected CD34 + cells generated during the reprogramming experiment were used. cDNA was synthesized from 1 μg RNA using the SuperScript II reverse transcriptase protocol (Thermo Fisher Scientific). RT-PCR was carried out using 5 μl of cDNA following the CTS CytoTune-iPS Sendai 2.1 Reprogramming Kit User Guide, and PCR products were analyzed on a 2% agarose gel.

To confirm the genomic integrity, a G-banding karyotype was performed from two clones. One clone free of virus genome and transgenes and with a normal karyotype was used for further expansion and full characterization from each line.

### GMP generation of hiPSC master cell banks (MCB) and working cell banks (WCB)

For the production of the MCB units under GMP conditions, the previously generated hiPSC clones were thawed and expanded in classified facilities, working in qualified clean rooms under an A-grade hood within a B-grade background using GMP-grade reagents, as described above. Previously, in compliance with internal risk analysis regarding previous production steps performed outside of the GMP environment, the hiPSC clone batches had been tested for sterility (BacTAlert, Biomerieux, Ph. Eur. 2.6.27), mycoplasma (qPCR, Ph. Eur. 2.6.7) and adventitious virus (cytopathic cultures). Afterward, two vials of the established homozygous compliant clones were thawed in two different laminin-coated 100-mm dishes, and after 5–7 days (70–80% confluence), each dish was split into 6–8 100-mm dishes. Upon reaching appropriate confluence again, the cells were collected and cryopreserved in 40–50 cryovials containing at least one million viable cells. After 24 h at − 80 $^\circ{\rm C}$, the cryovials were transferred to controlled gas phase liquid nitrogen tanks. For the production of the WCB, two MCB vials were thawed, expanded and frozen similarly to the MCB.

### Characterization

The characterization of hiPSCs was performed using the methodology previously described [21], with some modifications. The characterization includes immunocytochemistry (ICC) for pluripotency markers, alkaline phosphatase (AP) staining, differentiation in vitro by embryoid body (EB) formation and ICC and determination of short tandem repeats (STR).

A mycoplasma test was performed at early passages and before banking. Karyotype determination was repeated after banking.

#### Immunocytochemistry for pluripotency

To identify pluripotency markers, ICC was performed with antibodies against pluripotency factors (Nanog, OCT4, SOX2, TRA-1-81, TRA-1-60, SSEA3 and SSEA4) [21].

#### Differentiation by EB formation and ICC

In vitro differentiation was promoted by EB formation. After washing with PBS, hiPSC colonies were lifted with EDTA (Sigma-Aldrich, Art. No. E5134) and transferred to a 96-well plate in Essential 8 flex Medium using a multichannel pipette. The 96-well plate was centrifuged at 800 × g for 10 min and incubated at 37 $^\circ{\rm C}$ and 5% CO_2_ for 24 h. Early EBs were transferred to an ultralow attachment plate in Essential 8 flex Medium for an additional 24 h. Afterward, EBs were transferred to Matrigel (Corning, Art. No. 356234)-coated slide flasks and cultured in media to differentiate toward endo-, ecto- and mesoderm for 21–28 days. Cells were analyzed by ICC with specific antibodies against endodermal markers (a-fetoprotein (AFP) and forkhead box A2 (FOXA2)), ectodermal markers (bIII tubulin (TUJ1) and glial fibrillary acidic protein (GFAP)) and mesodermal markers (a-smooth muscle actin (ASMA) and gata binding protein 4 (GATA4)). Confocal images were taken using Leica TSC SPE/SP5 microscopes.

#### Characterization of MCB and WCB

Once the MCB was completed, the full characterization process was repeated as described above. After WCB manufacturing, only karyotyping and determination of STR were performed. A mean of two cell passages elapsed between the MCB and WCB.

#### MCB and WCB quality control

MCB and WCB were also screened for sterility (BacTAlert, Biomerieux, Ph. Eur. 2.6.27), mycoplasma (qPCR, Ph. Eur. 2.6.7), endotoxin (colorimetric, Ph. Eur. 2.6.14) and adventitious virus (cytopathic culture). Viability was assessed by flow cytometry dye exclusion (7AAD, Beckman) before freezing, and recovery after thawing was assessed by counting the number of colonies 5–6 days after thawing and plating.

## Results

Homozygous CB units that passed potency quality controls on a reference sample (in brief, CD34 + viability maintained and clonogenic potential proven) and for which donors had signed a specific informed consent were selected by highest HLA-match coverage for the Spanish population. Relevant genetic information of the homozygous CB units was verified by NGS retesting (Table 1).

**Table 1:** Information on the seven established homozygous haplotypes

Haploline	HLA-A	HLA-B	HLA-C	HLA-DRB1	HLA-DQB1	HLA-DPB1	ABO type	Sex
CD34 iPS1-Sv4F-B8	29:02	44:03	16:01	07:01	02:02	11:01	O +	XY
Hz 30-18-3 CBiPS2-Sv4F-D10	30:02	18:01	05:01	03:01	02:01	02:02/04:01	O +	XY
Hz 3-7-15 CBiPS3-Sv4F-E9	03:01	07:02	07:02	15:01	06:02	02:01/04:01	O +	XY
Hz 1-8-3 CBiPS4-Sv4F-F6	01:01	08:01	07:01	03:01	02:01	01:01/03:01	O-	XX
Hz 33-14-1 CBiPS6-Sv4F-H6	33:01	14:02	08:02	01:02	06:02	02:01P	O-	XX
Hz 24-7-15 CBiPS7-Sv4F-I12	24:02	07:02	07:02	15:01	06:02	04:01P	O +	XY
Hz 11-27-1 CBiPS8-Sv4F-J1	11:01	27:05	01:02	01:01	05:01	04:01/11:01	O +	XX

**Fig. 1:**
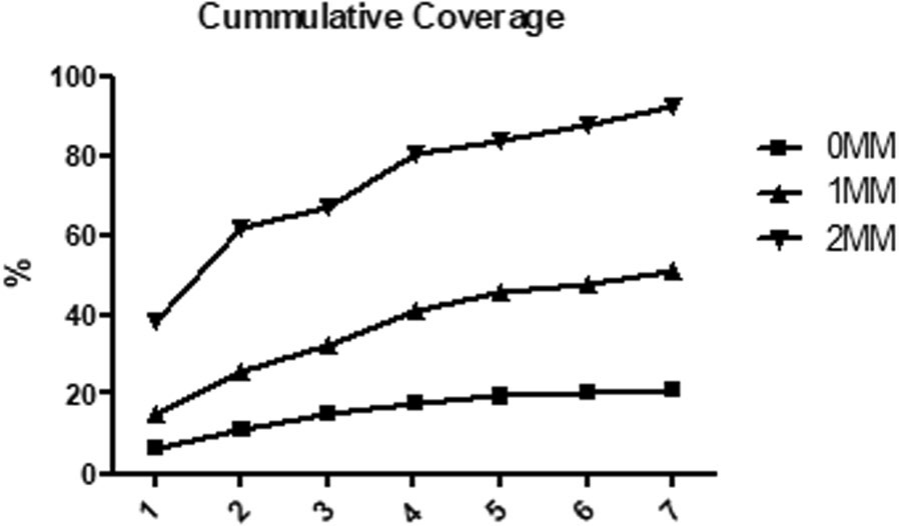
Cumulative coverage of haplolines. Cumulative coverage of the Spanish population with 7 haplolines considering no mismatch in HLA-A, HLA-B and HLA-DRB1 (0MM) or allowing mismatch in one of the three loci (1MM) or in two of them (2MM)

The actual cumulative coverage of the Spanish population for HLA match in loci HLA-A, HLA-B and HLA-DRB1 provided by the seven haplolines was calculated considering zero mismatch in the three loci or allowing mismatches in one or two loci, which resulted in 21.37%, 50.83% and 92.46% coverage, respectively (Fig. 1).

To set up the methodology of reprogramming blood cells extracted from frozen cord blood units, several experiments using CD34 + cells isolated from test units donated for research were performed. Reprogramming of CD34 + cells was performed with the Cytotune-iPS 2.0 Sendai Reprogramming Kit and CTS Cytotune-iPS 2.1 Sendai Reprogramming Kit under different multiplicities of infection (MOIs), and reprogramming efficiencies were compared. No significant changes in reprogramming efficiency were ascertained (Additional file 1: Table S1).

After thawing, isolation of the mononuclear fraction and immune magnetic selection of CD34 + cells from the homozygous cord blood units, the cells from all 7 samples could be expanded during 4 days in culture by a factor of 1.84 on average (see Table 2). A total of 1 × 10^4^ CD34 + cells were infected by a CTS Cytotune-iPS 2.1 reprogramming kit, and colonies emerged between 8 and 11 days after infection. The reprogramming efficiency was 1.04% on average.

**Table 2:** CB cell expansion and reprogramming efficiency

Name of iPSC clone	Hz 29-44-7 CBiPS1 Sv4F- B8	Hz 30-18-3 CBiPS2 Sv4F-D10	Hz 3-7-15 CBiPS3 Sv4F-E9	Hz 1-8-3 CBiPS4 Sv4F-F6	Hz 33-14-1 CBiPS6 Sv4F-H6	Hz 24-7-15 CBiPS7 Sv4F-I12	Hz 11-27-1 CBiPS8 Sv4F-J1
Alternative name	B8	D10	E9	F6	H6	I12	J1
Expansion of CD34 + cells, day1 (Number of cells)	0.23 × 10^6^	1.14 × 10^6^	0.66 × 10^6^	2.2 × 10^6^	0.63 × 10^6^	1.37 × 10^6^	0.61 × 10^6^
Expansion of CD34 + cells, day4 (Number of cells)	0.87 × 10^6^	1.65 × 10^6^	1.17 × 10^6^	3.46 × 10^6^	1.57 × 10^6^	2.76 × 10^6^	1.056 × 10^6^
Expansion factor	3.87	1.45	1.77	1.57	2.49	2.01	1.73
Reprogramming efficiency	0.86%	1.8%	1.07%	1.29%	0.45%	0.47%	1.34%

From each line, between 10 and 14 clones were manually picked and expanded, and cryotubes at low passages were frozen. Five clones per line were selected based on the visual appearance of the colonies and further expanded. From these 5 clones, cell pellets were prepared, RNA was extracted, and PCR was performed to prove the absence of signals derived from Sendai virus vectors (Additional file 1: Figure S1). From 2 clones of each line free of Sendai virus genome and transgenes, the karyotype was analyzed. Finally, 1 clone per line was selected for full characterization and banking.

The karyotype was euploid for all lines generated (3 lines 46, XX and 4 lines 46, XY) both at clone selection (passage 9–16) and at banking (passage 13–17). The pluripotency of all clones was confirmed by positive AP staining and by immunofluorescence analysis of the pluripotency-associated markers Nanog, OCT4, SOX2, TRA-1-81, TRA-1-60, SSEA3 and SSEA4. All 7 lines showed in vitro differentiation capacity toward the three germ layers, as proven by immunofluorescence-based detection of endoderm markers AFP and FOXA2, ectodermal markers TUJ1 and GFAP and mesodermal markers ASMA and GATA 4. hiPSC identity was confirmed by short tandem repeat analysis. STR markers were identical to the STR markers of the corresponding CD34 + cord blood cells.

All the lines have been registered at the Spanish National Stem Cell Bank. https://www.isciii.es/QueHacemos/Servicios/BIOBANCOS/BNLC/Paginas/LineasiPS.aspx, and at the Human Pluripotent Stem Cell Registry (ESi-093A – ESi-099A, https://hpscreg.eu/).

### Characterization of MCBs

**Fig. 2:**
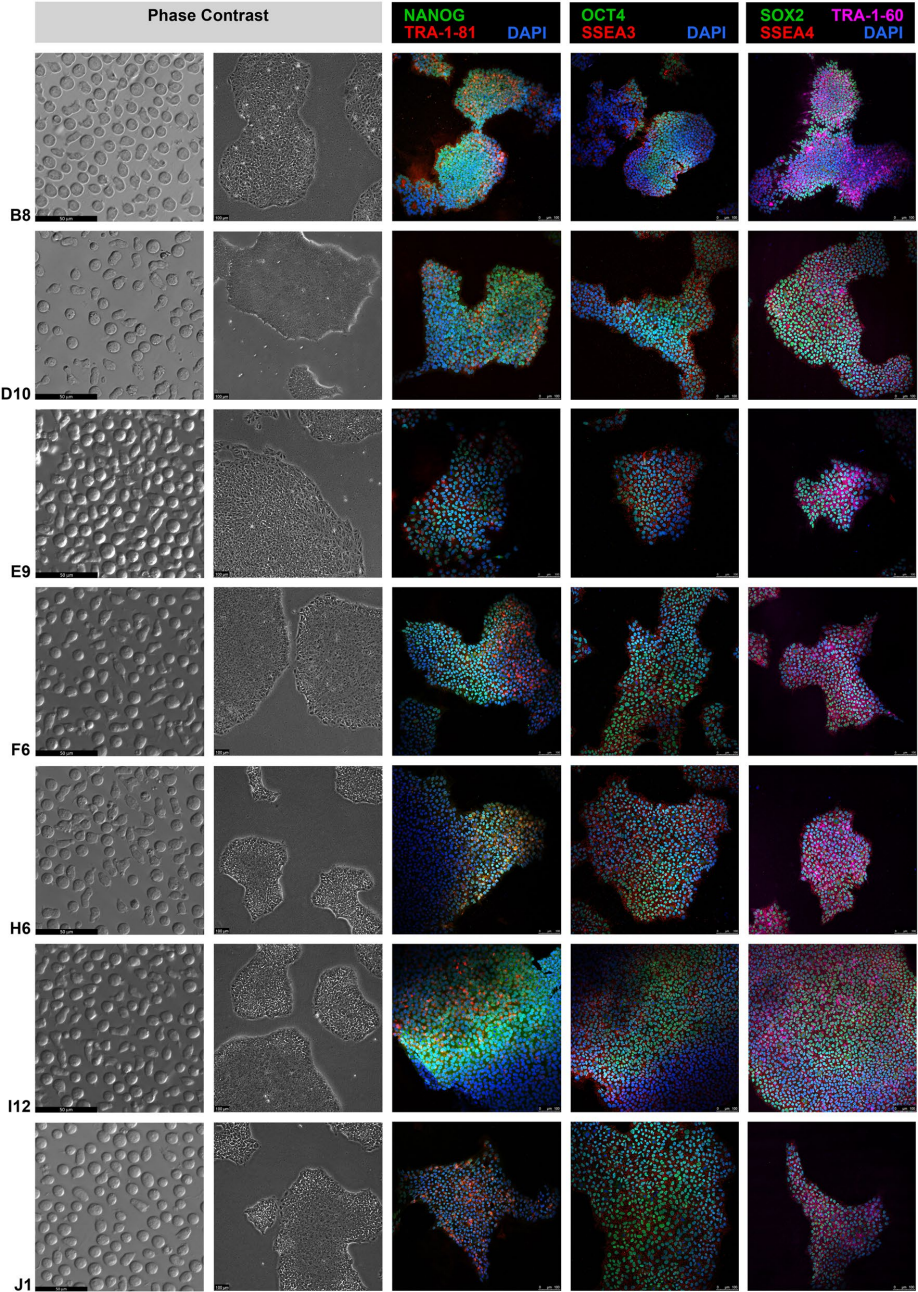
Morphology of CD34 + cells and generated iPSCs and ICC with pluripotency markers. Images showing phase contrast pictures of CD34 cells after expansion (first column), morphology of iPSC colonies at different passages (second column, B8: passage 19, D10: passage 18, E9: passage 14, F6: passage 15, H6: passage 16, I12: passage 10, and J1: passage 16), and confocal images of immunodetection of pluripotency markers (NANOG, OCT4, SOX2, TRA-1–81, TRA-1–60, SSEA3 and SSEA4, columns 3, 4, and 5; staining was performed at different passages of clones: B8, passage 22; D10, passage 21; E9, passage 19; F6, passage 17; H6, passage 17; I12, passage 17; and J1, passage 19.)

**Fig. 3:**
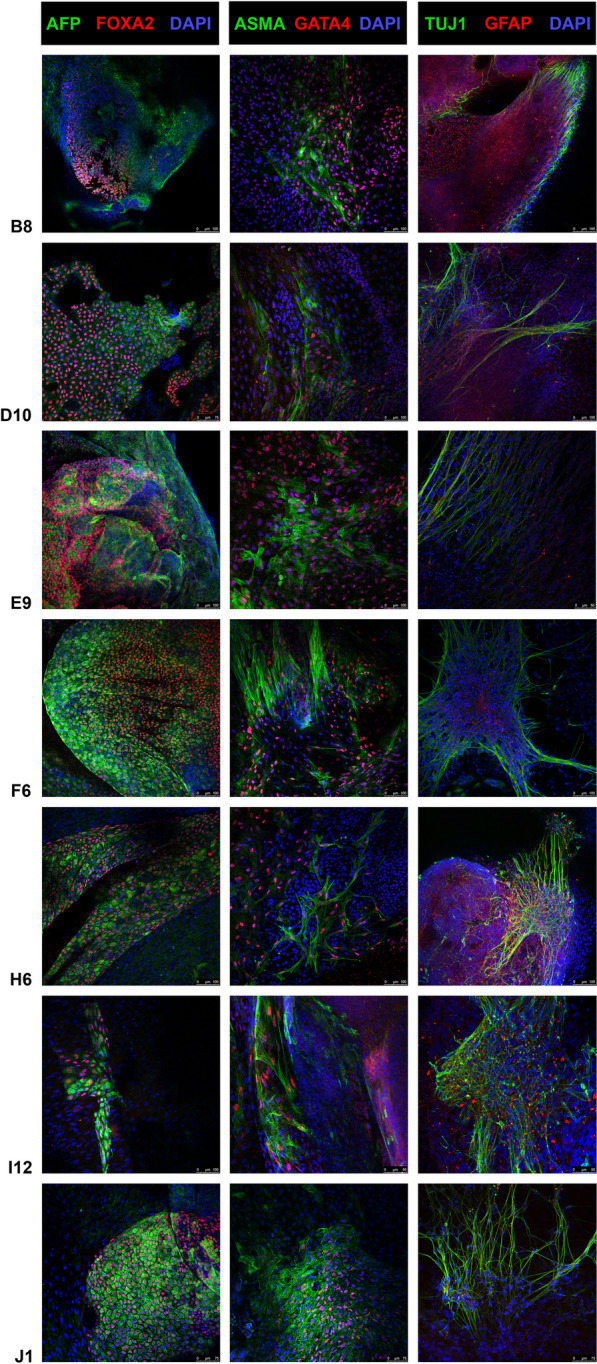
ICC with differentiation markers. ICC of in vitro differentiation of embryoid bodies using specific antibodies against the endodermal markers AFP and FOXA2 (left column), mesodermal markers ASMA and GATA4 (middle column), and ectodermal markers TUJ1 and GFAP (right column). Nuclei were stained with DAPI. Stainings were performed at different passages of clones: B8, passage 24; D10, passage 21; E9, passage 19; F6, passage 18; H6, passage 17; I12, passage 17; and J1, passage 19

After MCB manufacturing, a normal karyotype was maintained in all cases (Additional file 1: Figure S2). All lines stained positive for AP activity (Additional file 1: Figure S3), and the expression of the 7 pluripotency markers was also maintained (Fig. 2), as was the in vitro differentiation ability (Fig. 3). hiPSC identity was confirmed by STR analysis (Additional file 1: Table S2).

After WCB manufacturing, karyotypes and STR were analyzed again, and no changes were observed.

All MCB and WCB production complied with quality control acceptance criteria for sterility, cell number and viability (Table 3 and Additional file 1: Table S3).

**Table 3:** Quality control assays and acceptance criteria for GMP-MCB and GMP-WCB

Attribute	Acceptance criteria
Sterility (Ph. Eur. 2.6.27)	Negative
Mycoplasma (Ph. Eur. 2.6.7)	Negative
Endotoxin (Ph. Eur. 2.6.14)	< 5 EU/mL
Adventitious viruses (cytopathic culture)	Negative
Viability upon freezing (7AAD negative)	> 50%
Recovery 7 days upon thawing	> 20 colonies or 50% confluence
Total cell number before freezing (Neubauer)	> 1 × 10^6^cells/vial
Viability by dye exclusion cytometry (7AAD)	≥ 50% 7AAD^−^

## Discussion

Nakatsuji et al. [7] calculated that 30 carefully selected hiPSC lines would provide coverage to 82.2% of the Japanese population coinciding in the 3 loci (HLA-A, HLA-B and HLA-DRB1), and 90.7% of the population would be covered with 50 hiPSC lines. However, identifying these 50 potential donors would necessitate studying the HLA system of 24,000 individuals. Okita et al. [22] reported that 140 homozygous donors for HLA haplotypes would cover 90% of the Japanese population, requiring the screening of 160,000 potential donors. Similarly, Gourraud et al. [23] estimated that 26,000 donors of European-American ancestry and 110,000 donors of African-American ancestry would need to be screened to obtain hiPSCs representing the 20 most frequent HLA haplotypes and that these lines would provide coverage to 50% and 22% of these populations, respectively. All this confirms that relatively few donors, but very carefully selected, would allow the generation of hiPSC lines with a strong potential for clinical utility. Similar calculations have been established, e.g., for the Australian population [24], the Finnish population [25] and the Korean population in comparison with China, Japan and the West [9].

The World Marrow Donor Association (WMDA) estimates 256.006 cord blood units (CBU) preserved in cord blood banks in Europe and 798.372 units in the world (https://statistics.wmda.info/). Cord blood banks represent an excellent source of HLA-type, virus-screened young cells that make excellent starting material for generating homozygous hiPSC lines. Moreover, due to the young age of the donor, cord blood cells present the advantage of having negligible accumulated somatic mutations [26] and a young epigenetic age signature [27].

Bone marrow registries represent an alternative to CB banks as potential providers of samples for hiPSC generation, but both the availability, lower invasiveness and the easy access to samples in the latter are obvious advantages to be considered.

Few commercialized allogeneic clinical-grade hiPSC lines are currently available from private companies (Fuji-CDI, in Wisconsin USA, with haplolines of the 5 most common HLA types matching 35% of the US population), with nonexclusive license fees and restriction rights to develop and commercialize a product. Very few hiPSC lines for allogenic cell therapy are provided by public research organizations such as the NIH through RUCDR Infinite Biologics, Korea HLA-Typed hiPSC Banking (KHIB), and the Center for iPS Cell Research and Application (CiRA).

CiRA has generated a total of 27 hiPSC lines made from 7 donors (4 peripheral blood and 3 cord blood) who are homozygous for 4 of the most frequent HLA types in Japan (https://www.cira-foundation.or.jp/e/research-institution/ips-stock-project/homozygous.html). These lines cover approximately 40% of the Japanese population [28]. Some of these cell lines have already been used in hiPSC cell-based clinical cell therapies. In a recent report, Lee et al. [9] described the generation of hiPSC lines with the 10 most frequent HLA-homozygous haplotypes, which can match 41.07% of the Korean population. Comparative HLA analysis indicates that the lines are relevant to other Asian populations, such as Japan, with some limited utility in ethnically diverse populations, such as the UK. Similarly, Rim et al. [16] reported the generation of 13 homozygous GMP-grade hiPSC lines from blood and cord blood cells with selected homozygous HLA types from the Catholic Hematopoietic Stem Cell Bank of Korea.

Kim et al. [29] reported 22 GMP-compliant homozygous HLA-type iPSC lines deposited in the Korea National Stem Cell Bank, which cover HLA haplotype matching for 51% of the Korean population.

A European hiPSC collection to manufacture medicinal cell therapy products needs to be developed within a global organization to meet emerging scientific medical and industrial needs.

Recently, an initiative led by IDIBELL/PCMR-C coordinated by Anna Veiga has been funded by COST (European Cooperation in Science and Technology) (https://www.cost.eu/actions/CA21151). The aim of this COST action is to provide a framework for the generation of hiPSCs homozygous for frequent HLA haplotypes, compatible with a significant percentage of the population to be used for cell therapy. The data collection system for such lines will be incorporated in the European Pluripotent Stem Cell Registry (hPSCReg—https://hpscreg.eu/).

The project includes all the relevant stakeholders: immunology experts, cord blood banks, hiPSC generation and banking centers, manufacturing centers, regulatory bodies, national agencies, and ethics experts. The challenge will be approached essentially by networking with all the stakeholders, sharing knowledge, standardizing methodology and developing an educational training program for researchers.

HAPLO-iPS will pioneer new approaches that will foster the progress of haplo-selected hiPS generation by the development, implementation and exploitation of a registry with all the information for the benefit of patients.

The selected haplotypes for the generation of the hiPSC lines described here represent some of the most frequent HLA-A, HLA-B and HLA-DRB1 haplotypes in Spain, most of which are shared by other populations in Europe and North and South America. These 7 haplolines achieve HLA-A, HLA-B and HLA-DRB1 match cumulative coverage of 21.37% of the Spanish population. For some applications of hiPSC-derived cell therapies, a less stringent HLA match could be acceptable or manageable with immunosuppression. In these cases, allowing the mismatch in one or two of the three loci would widen the coverage to 50% or nearly the complete Spanish population with the 7 generated haplolines.

CBUs in CB banks have already undergone testing for viral blood pathogens, such as HIV, hepatitis B and C virus, malaria, Chagas disease and human T-cell lymphotropic virus I and II, and donor medical screening, fulfilling the first requirement as starting material to manufacture hiPSCs for clinical applications. The CBUs selected in this study were quality verified before reprogramming by thawing a segment and testing CD34 + and CD45 + content and viability and hematopoietic clonogenic capacity to verify the quality data at the prefreezing stage.

Use of hiPSC lines as a critical intermediate product for the manufacture of medicinal cell therapy products requires demonstration of comparability of lines derived from different individuals and in different facilities. This needs agreement on the quality attributes of such lines and the assays that should be used [10, 30–32].

Appropriate regulatory bodies, such as the Food and Drug Administration (FDA) in the US or the European Medicines Agency (EMA) in Europe, classify hPSC derivatives for cell therapy as advanced therapy medicinal products (ATMPs). This denotes, among other requirements, that PSCs must be produced under current Good Manufacturing Practice (GMP) conditions in facilities with a relevant product manufacturing license under strict quality assurance.

The generation of clinically acceptable hiPSCs includes donor selection, original cell sourcing, reprogramming methodology, culture and expansion, testing and banking. Although GMP-compliant hiPSCs have been generated [14–16, 33], some authors have adapted hiPSC lines established in standard conditions to culture in GMP conditions [17]. In our case, although the reprogramming process took place in a standard facility, the generation of MCBs and WCBs was performed in a GMP-certified facility. In fact, most ongoing current clinical trials are using hiPSCs generated in no-GMP conditions and then qualified for GMP by conversion in GMP culture conditions and additional testing [18].

The hiPSCs for clinical application should be developed under stringent ethical guidelines from anonymized traceable and tested donors who have signed an informed consent form [34]. IPS-PANIA donors were recontacted and invited to participate in the project and signed a new informed consent form, which was approved by the relevant ethics committee.

Since hiPSCs from MCBs and WCBs could have passage numbers of 20 or higher and it is well known that hiPSCs in long-term culture can incorporate de novo mutations [35], quality controls to ensure the genetic integrity of hiPSCs should be standardized [34, 36]. The International Society for Stem Cell Research (ISSCR) recently published the document “Standards for Human Stel Cell Use in Research” (https://isscr.org/standards-document), which provides recommendations for quality control of hiPSCs, including standards for assessing genetic integrity. A robust and standardized quality control (QC) system is needed for screening and testing hiPSCs for clinical purposes [31, 32, 37]. In our project, all the produced final cell products underwent testing by independent QC laboratories, including all mandatory analysis and safety assays that are commonly agreed upon in the field (Table 3 and Additional file 1: Table S3).

Here, we describe the methodology in which hiPSC haplolines intended to become an intermediate product of different medicinal products that have been generated with full traceability, using exclusively GMP-grade or clinical use approved reagents, and expanded and banked in clean rooms following a fully GMP-complying procedure. This approach is in line with the idea that iPSCs are not cell therapy medicines per se but a critical intermediate product. GMP manufacturing of ATMP allows certain flexibility in the application of GMP standards for starting material and intermediate cell products, as long as a risk-based analysis is applied and the necessary quality and safety control assays are performed.

Clinical medicine using iPS cells has advanced safely and effectively by making full use of current scientific standards, but tests on cell safety need to be further developed and validated [6, 10, 28, 35, 38, 39].

The generation of a collection of HLA-matched hiPSCs from more frequent haplotypes will avoid the need for the generation of specific hiPSCs for each patient, reducing time and costs. The need for immunosuppression after cell therapy would also be reduced [19]. This could lead to fewer medical complications and hospitalizations, lower mortality rates and better quality of life after the application of hiPSC-derived cell therapy.

HLA-matched hiPSC banks will offer clinical researchers off-the-shelf pluripotent cells for the quick, safe and easy production of derivatives needed for clinical application. These iPSC lines will be available for differentiation into multiple cell types for the treatment of ophthalmological, hematological, cardiac, neurological and metabolic diseases, among others.

### Conclusions

The haplobank described here constitutes a valuable intermediate cell bank to manufacture iPSC-derived cell therapies that can cover more than a fifth of the Spanish population by HLA matching and will also serve significant percentages of the European, North American and South American populations due to haplotype sharing. This bank will contribute to an international effort to create national haplobanks that can share haplolines, greatly expanding the possibilities of coverage of most of the population.

### Supplementary Information

**Additional file 1.** **Additional Figure 1.** Proof of absence of Sendai virus in generated iPSC clones PCR analysis of iPSC clones showing the absence of the SeV genome and transgenes. gDNA was extracted from cell pellets of clones, and PCR was performed following the instructions of the CTS Cytotune-iPS Sendai 2.1 Reprogramming Kit user guide (B6, passage 20, lane 2; D10, passage 19, lane 3; E9, passage 14, lane 4; F6, passage 16, lane 5; H6, passage 17, lane 6; I12, passage 11, lane 6; and J1, passage 17, lane 7. Positive control was applied at lane 1, negative controls on lines 9 (sample w/o RT), line 10 (sample w/o Sendai virus infection), and 11 (H_2_O).

## Data Availability

All the presented data are available for consultation.

## Abbreviations

ATMP: Advanced therapy medicinal product
BM: Bone marrow
CB: Cord blood
CBU: Cord blood unit
CTS: Cell therapy system
CTG: Clinical therapy grade
EMA: European Medicines Agency
EB: Embryoid body
FDA: Food and Drug Administration
GMP: Good Manufacturing Practice
hiPSC: Human-induced pluripotent stem cells
HLA: Human leukocyte antigen
ICC: Immunocytochemistry
iPSC: Induced pluripotent stem cell
MCB: Master cell bank
QC: Quality control
REDMO: Spanish Bone Marrow Donor Registry
STR: Short tandem repeats
WCB: Working cell bank
WMDA: World Marrow Donor Association
