# Supplementary material for: Generation of a bank of clinical-grade, HLA-homozygous iPSC lines with high coverage of the Spanish population
Source: Stem Cell Res Ther. 2023 Dec 13;14:366. doi: 10.1186/s13287-023-03576-1 (PMC10720139; doi:10.1186/s13287-023-03576-1)
Supplement: Supplementary file 1 — Additional file 1. Additional Figure 1. Proof of absence of Sendai virus in generated iPSC clones PCR analysis of iPSC clones showing the absence of the SeV genome and transgenes. gDNA was extracted from cell pellets of clones, and PCR was performed following the instructions of the CTS Cytotune-iPS Sendai 2.1 Reprogramming Kit user guide (B6, passage 20, lane 2; D10, passage 19, lane 3; E9, passage 14, lane 4; F6, passage 16, lane 5; H6, passage 17, lane 6; I12, passage 11, lane 6; and J1, passage 17, lane 7. Positive control was applied at lane 1, negative controls on lines 9 (sample w/o RT), line 10 (sample w/o Sendai virus infection), and 11 (H2O). [file 13287_2023_3576_MOESM1_ESM.docx]

**Additional results**

To get closer to conditions for clinical translation, different growth conditions for the expansion of CD34+ cells were compared, and the reprogramming of CD34+ cells was performed with the two different Invitrogen Cytotune-iPS reprogramming kits (2.0 and CTS 2.1) and two different multiplicities of infections (MOI) of 5:5:3 and 5:2,5:3.

(Additional Table 1).

**Additional Table 1. Expansion and reprogramming of CD34+ cells.**

| Experiment | No. of CD34+ cells | Cytotune kit | MOI | Reprogramming efficiency |
| --- | --- | --- | --- | --- |
| 1 | 0.23x10^6^  After 3 days:  0.87x10^6^ | 2.0 | 5:5:3 | 1.0% |
| 2 | 0.23x10^6^  After 3 days:  0.87x10^6^ | 2.1 | 5:5:3 | 0.86% |
| 3 | 1.4x10^6^  After 3 days:  1.65x10^6^ | 2.1 | 5:5:3 | 1.9% |
| 4 | 1.4x10^6^  After 3 days:  1.65x10^6^ | 2.1 | 5:2.5:3 | 1.8% |
| 5 | 0.66x10^6^  After 3 days:  1.17x10^6^ | 2.1 | 5:2.5:3 | 1.07% |
| 6 | 2.2x10^6^  After 3 days:  3.46x10^6^ | 2.1 | 5:2.5:3 | 1.29% |

Reprogramming of CD34+ cells. Experiments were carried out to set up and optimize the methodology. Columns showing the quantity of extracted CD34+ cells after their extraction and after 3 days of incubation in SP34 SFM supplemented with cytokines, the Sendai virus kit used for transduction of CD34 cells, the multiplicity of infection (MOI) and the efficiency of reprogramming.

**Additional Table 2. STR analysis.** DNA fingerprinting profiles of CD34+ cells extracted from cord blood units (first row in each table), iPSC lines generated from them (second row), and from MCBs and WCBs (third row and fourth row, respectively).

See table in separate file.

**Additional Table 3. MCB and WCB number and viability release assay data.**

| iPSC line | Prefreeze viability (%) | No. of frozen cells | Post-thaw No. of colonies/vial |
| --- | --- | --- | --- |
| MCB CD34 iPS1 Sv4F-B8 | 96.3 | 1.0 x 10^6^ | >7.000 |
| MCB Hz 30-18-3 CBiPS2 Sv4F-D10 | 73.93 | 1.0 x 10^6^ | >2.500 |
| MCB Hz 3-7-15 CBiPS3 Sv4F-E9 | 78.24 | 1.0 x 10^6^ | >5.000 |
| MCB Hz 1-8-3 CBiPS4 Sv4F-F6 | 89.51 | 1.6 x 10^6^ | >2.000 |
| MCB Hz 33-14-1 CBiPS6 Sv4F-H6 | 92.79 | 1.6 x 10^6^ | >6.000 |
| MCB Hz 24-7-15 CBiPS7 Sv4F-I12 | 90.03 | 1.2 x 10^6^ | >3.000 |
| MCB Hz 11-27-1 CBiPS8 Sv4F-J1 | 97.63 | 1.0 x 10^6^ | >2.000 |
| WCB CD34 iPS1 Sv4F-B8 | 95.02 | 1.75 x 10^6^ | >7.000 |
| WCB Hz 30-18-3 CBiPS2 Sv4F-D10 | 95.20 | 1.4 x 10^6^ | >2.500 |
| WCB Hz 3-7-15 CBiPS3 Sv4F-E9 | 90.11 | 1.73 x 10^6^ | >4.000 |
| WCB Hz 1-8-3 CBiPS4 Sv4F-F6 | 93.91 | 1.6 x 10^6^ | >1.500 |
| WCB Hz 33-14-1 CBiPS6 Sv4F-H6 | 95.18 | 1.1 x 10^6^ | >1.000 |
| WCB Hz 24-7-15 CBiPS7 Sv4F-I12 | 95.57 | 1.3 x 10^6^ | >3.000 |
| WCB Hz 11-27-1 CBiPS8 Sv4F-J1 | 93.06 | 1.1 x 10^6^ | >1.500 |

**Additional Figure 1. Proof of absence of Sendai virus in generated iPSC clones.**


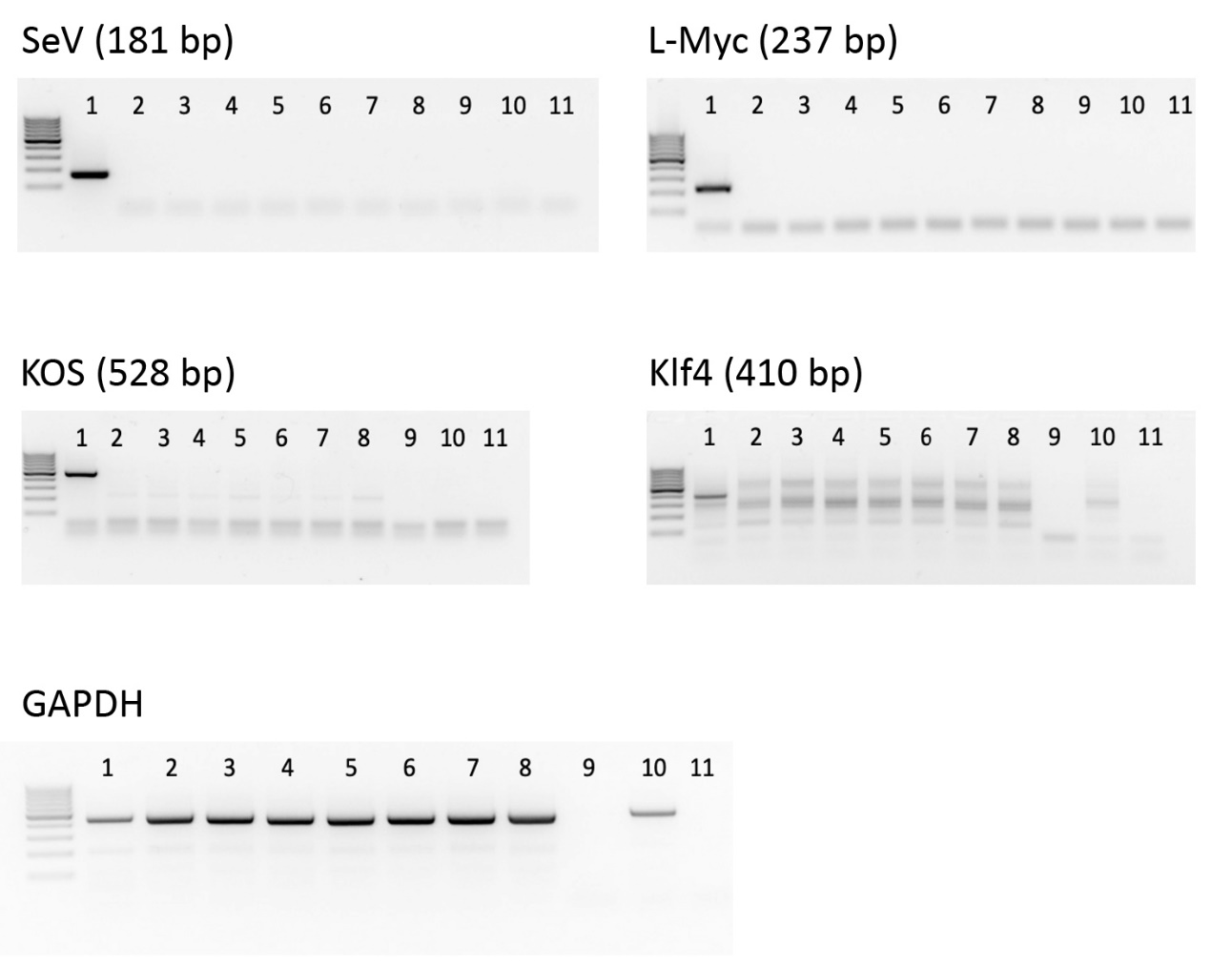


PCR analysis of iPSC clones showing the absence of the SeV genome and transgenes. gDNA was extracted from cell pellets of clones, and PCR was performed following the instructions of the CTS Cytotune-iPS Sendai 2.1 Reprogramming Kit user guide (B6, passage 20, lane 2; D10, passage 19, lane 3; E9, passage 14, lane 4; F6, passage 16, lane 5; H6, passage 17, lane 6; I12, passage 11, lane 6; and J1, passage 17, lane 7. Positive control was applied at lane 1, negative controls on lines 9 (sample w/o RT), line 10 (sample w/o Sendai virus infection), and 11 (H_2_O).

**Additional Figure 2. Karyotype analysis of iPSC clones.**

**
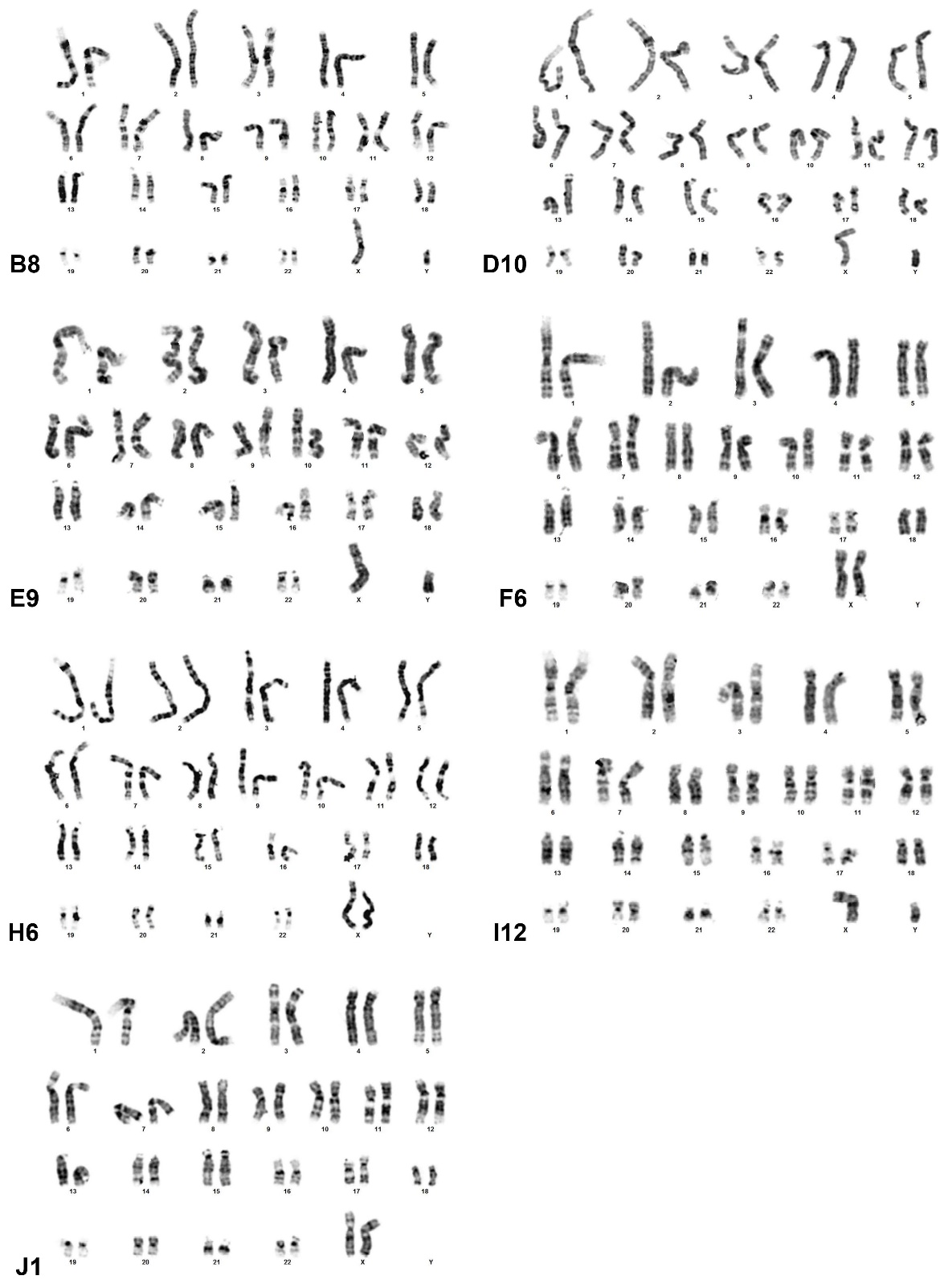
**

G-Banding **k**aryotypes of representative metaphases showing diploid 46, XX or XY karyotypes without any detectable abnormalities. (Karyotypes were performed at different passages: B6, passage 22; D10, passage 21; E9, passage 20; F6, passage 17; H6, passage 18; I12, passage 17; and J1, passage 18).

**Additional Figure 3. AP activity staining of clones.**

**
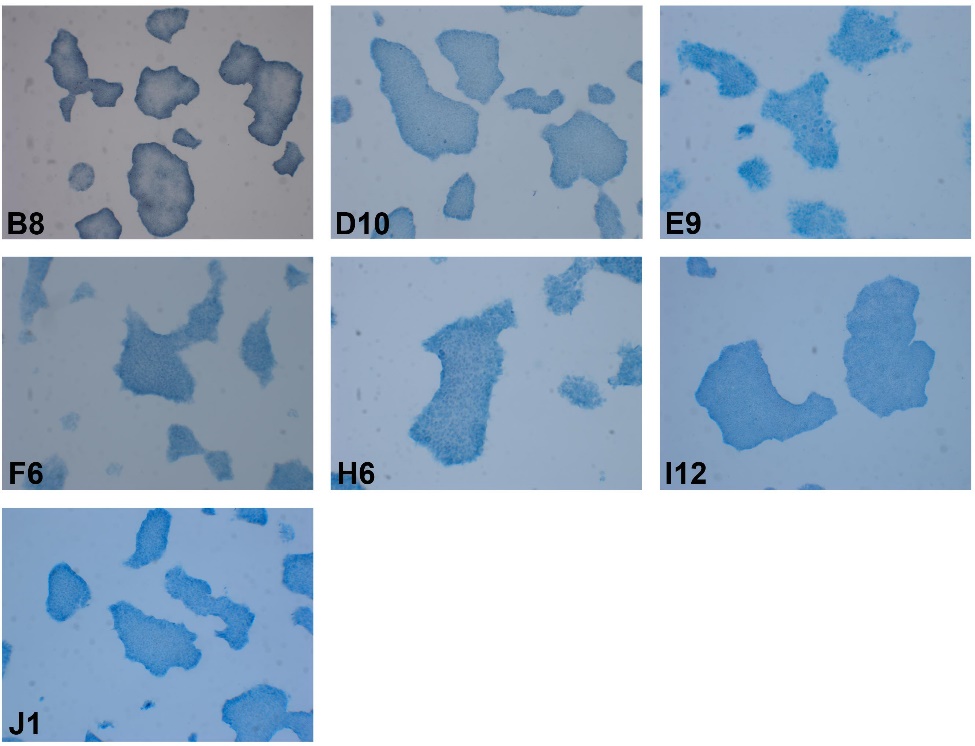
**

AP staining was performed at different passages of clones: B8, passage 23; D10, passage 21; E9, passage 19; F6, passage 17; H6, passage 17; I12, passage 17; and J1, passage 19.
